# Contact Networks in a Wildlife-Livestock Host Community: Identifying High-Risk Individuals in the Transmission of Bovine TB among Badgers and Cattle

**DOI:** 10.1371/journal.pone.0005016

**Published:** 2009-04-29

**Authors:** Monika Böhm, Michael R. Hutchings, Piran C. L. White

**Affiliations:** 1 Environment Department, University of York, York, United Kingdom; 2 Disease Systems, Scottish Agricultural College (SAC), Edinburgh, United Kingdom; University of California Merced, United States of America

## Abstract

**Background:**

The management of many pathogens, which are of concern to humans and their livestock, is complicated by the pathogens' ability to cross-infect multiple host species, including wildlife. This has major implications for the management of such diseases, since the dynamics of infection are dependent on the rates of both intra- and inter-specific transmission. However, the difficulty of studying transmission networks in free-living populations means that the relative opportunities for intra- versus inter-specific disease transmission have not previously been demonstrated empirically within any wildlife-livestock disease system.

**Methodology/Principal Findings:**

Using recently-developed proximity data loggers, we quantify both intra-and inter-specific contacts in a wildlife-livestock disease system, using bovine tuberculosis (bTB) in badgers and cattle in the UK as our example. We assess the connectedness of individuals within the networks in order to identify whether there are certain ‘high-risk’ individuals or groups of individuals for disease transmission within and between species. Our results show that contact patterns in both badger and cattle populations vary widely, both between individuals and over time. We recorded only infrequent interactions between badger social groups, although all badgers fitted with data loggers were involved in these inter-group contacts. Contacts between badgers and cattle occurred more frequently than contacts between different badger groups. Moreover, these inter-specific contacts involved those individual cows, which were highly connected within the cattle herd.

**Conclusions/Significance:**

This work represents the first continuous time record of wildlife-host contacts for any free-living wildlife-livestock disease system. The results highlight the existence of specific individuals with relatively high contact rates in both livestock and wildlife populations, which have the potential to act as hubs in the spread of disease through complex contact networks. Targeting testing or preventive measures at high-contact groups and individuals within livestock populations would enhance the effectiveness and efficiency of disease management strategies.

## Introduction

Most pathogens which are of concern to humans and their livestock are generalist in nature and thus able to cross-infect multiple host species. For example, 77% of pathogens of livestock have been found to affect more than one host species [1]. When disease exists in multiple host systems, its dynamics are further complicated depending on the rate of inter-specific disease transmission [2], [3]. Theoretical models suggest that where strong spatial segregation leads to distinct sub-groupings within a population, as is the case for territorial species, inter-species transmission may be the dominant transmission pathway, so that the presence of an alternative host is required for pathogen establishment [2], [4]. This conclusion has major implications for disease management. However, the difficulty of studying contact networks in free-living populations means that the relative rates of intra- versus inter-specific disease transmission have not previously been demonstrated empirically within any wildlife-livestock disease system.

Disease transmission within populations can follow complex contact patterns, based on differences in behaviour between host individuals, such as due to their relative positions in a social hierarchy [5]. Failure to account for the complexity of social networks through which diseases may be transmitted may in turn be responsible for the failure of disease control strategies based simply on one-off population reduction [6]. For example, a study of possums *Trichosurus vulpecula* Kerr in New Zealand showed a non-linear relationship between contact rates and population density [7], clearly in contrast to the traditional assumption of density dependence. Moreover, the influence of social hierarchy on disease dynamics becomes relatively more important at low disease prevalence [8], with consequent implications for our ability to eradicate disease in many wildlife hosts. Improving our understanding of the behavioural processes underlying transmission events is likely to provide valuable insights into the nature of real-life contact networks and help to refine management strategies aimed at reducing the frequency of contacts between hosts and hence the rate of transmission.

Contact network structure can have major implications for the dynamics of infections [9]–[12]. The persistence of epidemics within a social network relies on population mixing at two levels: large levels of mixing within distinct social groups (local mixing) and occasional mixing with individuals outside the social group (global mixing) [13]. Studies of contact networks for livestock populations, concentrating on between-farm movements, have highlighted considerable heterogeneity between and within contact networks [14]. For example, for direct-contact diseases of cattle, 20% of holdings can contribute at least 80% of new cases of an infection [9]. Within such a heterogeneous contact network, management targeted at the highly connected nodes should be extremely effective and is likely to be much more efficient than untargeted mass control [15]–[17].

The effective management of a livestock disease however, also depends on an understanding of the networks occurring at the farm level (i.e. within-herd). For livestock diseases which have wildlife hosts, additional consideration needs to be given to the contact network within the wildlife host and between the livestock and wildlife. Quantifying multi-species contact networks in terms of the degree of connectedness between individuals at both intra- and inter-specific levels may help to pinpoint ‘high-risk’ individuals or groups of individuals and, ultimately, lead to more accurate predictions of disease dynamics. However, quantification of contact networks in wildlife has been difficult if not impossible, due to limitations of available technology. Direct observations of captive populations and observations of wild animals at feeding stations, den sites or in open habitats have often been used to study direct interactions (e.g. [18]–[21]), but their findings are of limited applicability to wild populations or are location-specific. Radio-telemetry studies, although useful indicators of the extent of interaction patterns [5], [22], [23], operate at too coarse a resolution to determine contact rates accurately.

Here, we use proximity data-logging devices to quantify both intra-and inter-specific direct contacts in a wildlife-livestock disease system for the first time. Proximity data loggers have been used for monitoring intra-specific contact behaviour in possums, raccoons *Procyon lotor* L. and cattle [7], [24], [25], but have never been employed previously in a multi-species context.

We use bovine tuberculosis (bTB) in badgers *Meles meles* L. and cattle in Britain as our disease system. The persistence of bTB in cattle in Britain has been assisted by the presence of wildlife hosts, principally the badger, for *Mycobacterium bovis* Karlson & Lessel, the causative agent of the disease [26]. Cattle movements are the most important factor determining the irruption of bTB in areas outside traditional disease hotspots [27], [28], and a core part of bTB management strategies is therefore based on minimising contact between herds. In high-risk areas, cattle movements also play a role, but unidentified local-scale processes may account for up to 75% of unexplained variation in the incidence of cattle bTB [29]. The spread of bTB in new areas and its persistence in existing hotspot areas depend on the existence of a suitable host community. A greater understanding of transmission opportunities among badgers and between badgers and cattle would provide valuable information for the management of the disease.

Despite considerable research on bTB in cattle and badgers since the 1970s, the transmission process to cattle remains poorly understood, although the evidence suggests that the infection is spread by airborne transmission [30]. Based on visual observations, it has been suggested that badgers generally avoid close contact with cattle on pasture [31]. Previous authors have presumed therefore that most cases of bTB in cattle that have their source in badgers arise through inspiration of bacilli during grazing of grass contaminated with infected badger urine, sputum or faeces [32], [33]. This is most likely to occur when cattle graze around badger excretory products at latrines and crossing-points, where badgers may urinate or defecate after passing through hedgerows [34]. However, more recent research has highlighted the potential importance of both indirect and direct badger-cattle contacts at cattle feeding troughs and within and around farm buildings [35], [36]. Visual observations inevitably account for only a small proportion of the total contacts an individual makes, and it is possible that direct contacts between badgers and cattle on pasture have been underestimated previously. Transmission of bTB among badgers is thought to occur predominantly via inhalation of aerosols [37], since the majority of lesions in badgers occur in the lungs or thoracic lymph nodes [38], [39]. The potential importance of close direct interactions among hosts means that monitoring such interactions is of fundamental importance in understanding the opportunities for transmission of the infection. Proximity data-loggers provide us with the opportunity to quantify these contacts in considerable detail for the first time.

Across much of its range in Britain, the badger lives in highly territorial social groups and is, therefore, expected to show a largely local contact structure where populations are undisturbed. It has recently been shown that spatial segregation caused by territoriality led to highly localised and stable infections, which were restricted to particular territories [40]. Similarly, cattle may form sub-groupings and dominance hierarchies within the herd [41]. Based on this and previous research on badger-cattle interactions, we therefore expect to find (1) a largely heterogeneous direct contact network within the cattle herd based on sub-groupings and hierarchies, (2) a heterogeneous contact network for badgers with a high level of contacts between individuals within the same group but considerably fewer interactions between individuals from different groups, and (3) very infrequent inter-species contacts between badgers and cattle.

## Materials and Methods

### Study site

**Figure 1:**
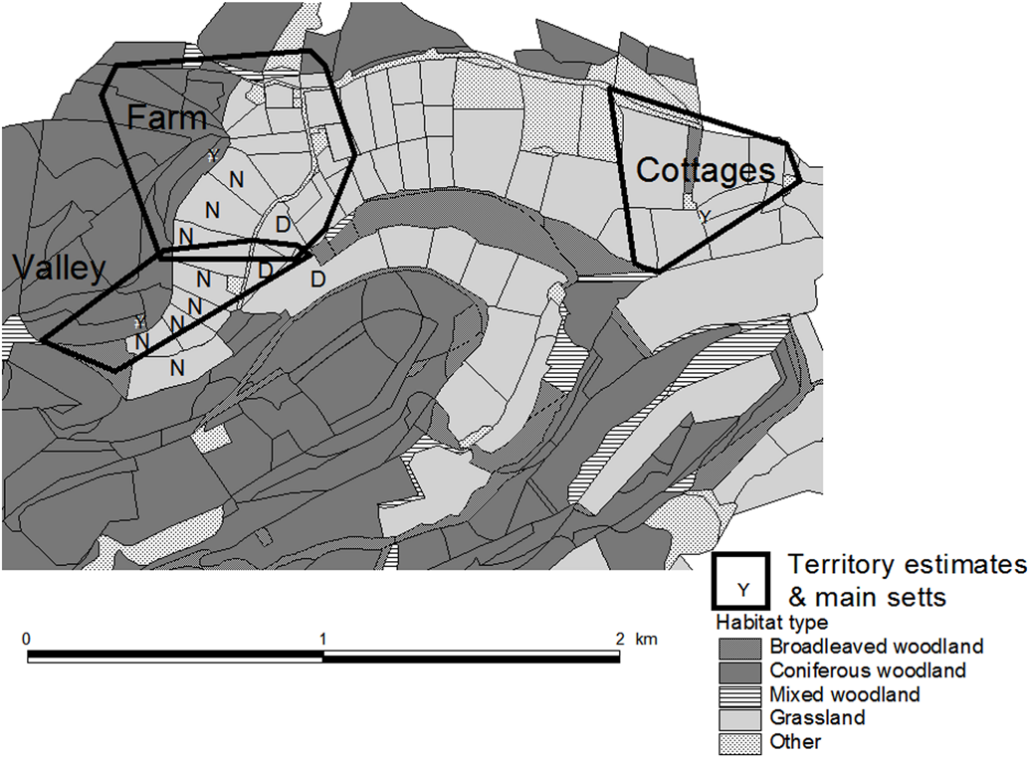
Dalby Forest study site. The main sett (den) and territories of the three badger social groups under study, as well as broad habitat categories, are shown. Territory estimates were derived from bait-marking returns (Valley & Cottages group) and radio-tracking (Farm). Both Farm and Valley groups overlap greatly with the grazing pasture of the study dairy herd (D, day pasture; N, night pasture).

The study was carried out at a site of approximately 4 km^2^ situated in Dalby Forest (North York Moors National Park, north-east England), a predominantly commercial coniferous plantation, although our study site at the north-eastern edge of the forest mainly consisted of pasture for livestock grazing and agricultural fields (Fig. 1). The main focus of our study was a dairy farm with a herd of approximately 80 cattle at any given time, the night grazing pasture of which overlapped extensively with the territories of two neighbouring badger social groups (Valley and Farm). All our study cows were housed outside over night, albeit with some restricted access to the farmyard, so that any contacts recorded between badgers and cattle would either occur in the pasture or the farmyard. A third badger social group (Cottages) was also under study, although this group was not immediately adjacent to the other groups (Fig. 1).

Badger social groups in Dalby Forest range from 3–7 adults per group [42]. Bait-marking returns estimated the group sizes of the Valley and Cottages groups at 5 and 6 adults respectively (using a dung pit index as described in [43]), while trapping throughout 2006 indicated group sizes of 3 (Farm), 4 (Valley) and 6 adults (Cottages) respectively.

### Badger trapping and collaring

From May to November 2006, badgers were trapped under UK Home Office and English Nature licences in cage traps baited with a mixture of badger food (containing peanuts, locust beans and flaked maize; CJ WildBird Foods Ltd., Shrewsbury, UK) and golden syrup. Cage traps were set in late afternoon and checked at dawn the following morning after a pre-baiting period of at least five days. All newly-captured individuals (apart from cubs, which were released immediately) were weighed and subsequently anaesthetised using a mixture of Vetalar (ketamine hydrochloride; Pharmacia & Upjohn), Domitor (medetomidine hydrochloride; Orion) and Torbugesic (butorphanol; Fort Dodge). Each badger was sexed, aged as either an adult (more than two years of age) or yearling (between one and two years of age; ageing was based on tooth wear as described in [44]), examined for signs of reproduction, injuries and parasites and then given a unique tattoo identification code in the inguinal region. We then fitted each badger with a collar holding a proximity logger. Once the collars were fitted, the animals were given Antisedan (atipamezole hydrochloride; Orion) as a reverse anaesthetic and left to recover from the anaesthetic before being released at the point of capture. Due to the finite amount of memory space on the proximity logger, regular trapping was carried out throughout the summer to recapture individuals for data download. Since the data download required the proximity logger to be physically linked via an interface box to a laptop, the procedure again required the administration of an anaesthetic. To minimise the negative impact caused by repeated anaesthesia, individuals were only considered for the download procedure if a month or more had passed since the previous administration of anaesthetic, while only subjecting individuals to a maximum of four procedures during the course of the study.

### Cattle collaring

At any one time, seven to eight randomly selected dairy cattle (∼10% of the herd) were fitted with proximity data loggers. Collar fitting and removal for data download were generally carried out during afternoon milking. Data were downloaded in the laboratory and collars were put back on the same individuals wherever possible. Where an individual was removed from the herd for production reasons (they were usually housed within the farmyard stables and no longer turned out onto pasture), a new individual was selected.

### Proximity data loggers

Proximity data loggers were supplied by Sirtrack Ltd (Havelock, New Zealand [24]). These transmit unique identification codes via a UHF transceiver whilst simultaneously receiving code signals from other loggers within detection range at 1.5 second intervals [24]. Once another data logger is detected, contacts continue until the receiving logger fails to detect the signal within a specified ‘separation time’, in our case fifteen seconds, the lowest setting possible (i.e. two detection events <15 seconds apart would constitute one contact and two detection events >15 seconds apart would constitute two contacts). The resulting information in terms of the ID of the data logger contacted, the date and start time of the contact, and its duration are then stored in the logger's memory. Each logger also emits a VHF signal transmitting at 173 MHz, which allows collars to be located in the field.

Although under ideal conditions, two data loggers that come into contact should record precisely the same information, the proximity loggers cannot achieve absolute spatial precision, due to radio-waves being reflected, refracted and absorbed by a number of natural features (vegetation, water bodies, height of ground, terrain etc.) under field conditions [25], [45]. This is especially true in those cases where the species under study are of different heights. Vertical detection distances between two proximity loggers caused by height differences, such as those between badgers and cattle, were found to be slightly larger than horizontal detection distances in laboratory trials [24]. The detection range of each logger can be adjusted using the appropriate UHF output power setting for the species under study, in order to overcome the problems posed by the size difference between cattle and badgers. UHF power settings range up to UHF 62, which is the shortest detection distance available. Since it has been shown that at UHF power settings of 57 and above, collars often failed to record contacts [46], we chose a detection distance of UHF 40 for the badger proximity loggers. This setting translated into detection distances of up to 4 m during laboratory trials. In field trials, however, a UHF power setting of 40 corresponded to an average contact initiation distance of 1.69±0.11 m and a contact termination distance of 2.74±0.12 m [46]. It also minimised the amount of one-second contacts recorded, which would otherwise lead to a shortage of memory space. Since these are most likely to occur when individuals are at the edge of the detection range [24], all one-second contacts were removed prior to data analysis. Previous work has shown that ∼28% of all contacts recorded are one-second contacts at UHF 45 [46].

Cattle proximity loggers were set to a lower power setting (50) to avoid them filling up too quickly with contacts during times when cows closely aggregated, such as during milking periods. This power setting translated into a more or less continuous detection range of 1.6 m in laboratory trials, although occasional longer distance contacts of up to 3.2 m were recorded. In field trials, this setting recorded an average contact initiation distance of 1.36±0.18 m and an average contact termination distance of 2.61±0.23 m [46]. The cattle loggers therefore covered approximately the same range as the badger loggers. At these settings, cattle and badger proximity loggers detected each other at distances of up to 3.5 m in the laboratory, with occasional intermittent detection of up to 4 m. Based on the relative results for individual loggers in the laboratory and the field described above, cattle-badger logger detection distances in the field would be expected to range between 1.5–2.5 m.

Badger proximity loggers were mounted onto a standard leather collar weighing 150 g in total (approximately 1.5–1.8% of badger body mass). Cattle proximity loggers were housed together with a replaceable C cell battery in a plastic casing, which was mounted onto adjustable collars made from synthetic belting with a plastic clip for easy fitting and removal.

### Data sorting

All trapping nights were removed from the badger contact data set. Trapping nights were defined as starting from the average monthly emergence time on the evening the traps were set (calculated from emergence times of Dalby badgers recorded during a three-year radio-tracking study; M. Böhm, unpublished data), and lasted until 12:00hrs the following day, which corresponds to the average release time of badgers that underwent anaesthetic procedures. If any badger that was fitted with a data logger was caught, we excluded all information from all collared badgers of the same and neighbouring group (Farm and Valley badgers only) until 12 noon the day following its release back into the wild. This 24-hour buffer helped us to avoid data collection during periods of abnormal behaviour caused by the handling procedure and allowed badgers undergoing anaesthetic procedures to fully recover and acclimatise to the collar. As a result, we collected badger contact data throughout nine sampling periods (Table 1).

**Table 1:** Sampling periods for badgers, showing the groups studied and the number of individuals collared.

Sampling period	From	Until	Groups	No. of collared badgers at start of period
1	11 May	14 May	Cottages	3
2	16 May	21 May	Cottages	3
3	23 May	5 June	Valley, Cottages	3, 4
4	8 June	12 June	Valley, Cottages	3, 5
5	15 June	26 June	Valley, Cottages	4, 3
6	28 June	3 July	Valley, Cottages, Farm	4, 3, 2
7	4 July	24 July	Valley, Cottages, Farm	4, 3, 2
8	26 July	22 August	Valley, Cottages, Farm	4, 3, 2
9	24 August	18 September	Valley, Cottages, Farm	4, 4, 2

Acclimatisation buffers of 24 hrs were also applied to the cattle data after each collaring event, starting from the end of the afternoon milking period at 16:30GMT to the end of afternoon milking the following day. Cattle data were collected throughout four sampling periods (Table 2). The nine sampling periods for badgers were subsequently aggregated to four periods for comparison to cattle as shown in Table 2.

**Table 2:** Sampling periods for cattle, showing the number of individuals collared and the corresponding badger sampling periods.

Sampling period	From	Until	No. of collared cattle	Corresponding badger sampling periods
1	19 May	5 June	7 (5)	2, 3
2	7 June	26 June	7 (6)	4, 5
3	28 June	18 July	7 (6)	6, 7
4	11 August	3 September	8 (4)	8, 9

Numbers in brackets denote the number of incomplete cattle records due to technical problems as described in results section.

On several occasions, badgers from the Cottages group and some cattle lost their collars in the field. In these circumstances, data were removed from the analysis from the time at which the collar was last known to be still attached. For badgers, this was determined by regular radio-tracking.

### Measures of connectedness

In the following analysis, “intra-group” refers to contacts within a badger social group, “intra-herd” refers to contacts within the cattle herd, “inter-group” refers to contacts between badgers from neighbouring social groups (Farm and Valley groups only) and “inter-species” to badger-cattle contacts (cattle contacts with badgers from Farm and Valley groups).

### Intra-group and intra-herd connectedness

To standardize values between individuals, we calculated the daily contact frequency *C*_freq_ and the daily contact duration *C*_dur_ (in seconds) by dividing the total number of contacts and the total contact duration by the number of days for which each individual's proximity logger was attached. We then divided the resulting daily estimates by the number of individuals available for contact at any one time (i.e. the number of other individuals within the group wearing loggers at the time).

For each individual we also calculated the average duration per contact *AV*_dur_ (in seconds) by dividing the total duration of contacts by the total number of contacts, as well as average (*AV*_int_) and maximum time interval (*MAX*_int_) between successive contacts. *AV*_int_ and *MAX*_int_ were calculated from the initiation time of successive contacts to avoid ‘negative’ times where individuals were in contact with more than one individual at a time and contacts overlapped; both were weighted by the number of other individuals wearing proximity loggers within the group at the time. We calculated each connectedness measure as a total for the entire study period and for each of the four sampling periods respectively. Prior to further analysis, connectedness measures were log10-transformed to fit a normal distribution. All analyses were carried out separately for badger and cattle data.

We tested for correlations between all five connectedness measures (*C*_freq_, *C*_dur_, *AV*dur, *AV*_int_, *MAX*_int_) using Pearson's product moment correlations to assess the consistency in measurements of individual proximity loggers and to define uncorrelated connectedness measures for inclusion in our analysis. This is of particular importance for the calculation of a connectivity index (see below) since the use of correlated measures essentially includes a related property twice in the index, thus potentially overemphasizing the importance of an individual within the network. The results of this analysis are presented in the supporting information (Text S1; Fig. S1 & S2).

We then assessed differences between the remaining uncorrelated connectedness measures for all intra-group and intra-herd contacts using linear mixed-effects models (LME) which allow analysis of hierarchically structured data (i.e. including nested factors and/or repeated measures [47]). We sampled connectedness measures repeatedly across individuals and the four sampling periods, allowing us to treat these factors as random effects by assigning them as nested grouping factors thereby helping to reduce the number of unknown regression components in the model [47]. A first order autoregressive structure was used to overcome potential time-dependence of the data between successive sampling periods. The models were implemented in Brodgar v2.5.1 (Highland Statistics Ltd, Newburgh, Scotland) and were fitted using restricted log-likelihood (REML). We assessed the importance of sex, age, social group and sampling period on connectedness measures in the badger model, and of sampling period only in the cattle model. Formal statistical comparison between cattle and badger contact patterns was not possible, since the detection distances were set slightly differently for cattle and badger proximity loggers. However, our laboratory and field trials of logger detection distances suggest that the results from the different loggers should be approximately comparable.

Finally, we produced an intra-group/intra-herd connectedness index *CI*, which allowed us to rank order our study animals in terms of their connectedness within the intra-group/intra-herd network. For this, we ranked each of the remaining uncorrelated connectedness measures in turn from low connectedness to high connectedness and summed the rankings for each individual (we used ranking scores from 1–12 for badgers and 1–13 for cattle). Low connectedness referred to small number of contacts, short contact durations and long time intervals between successive contacts, while high connectedness referred to large number of contacts, long contact durations and short time intervals between successive contacts.

### Inter-group/inter-species connectedness

The Cottages group was excluded from the analysis of inter-group and inter-species contacts due to its location. To assess connectedness of individuals, we calculated three of the connectedness measures described above, *C*_freq_, *C*_dur_ and *AV*_dur_. We again weighted *C*_freq_ and *C*_dur_ by the number of inter-group and inter-species contacts available at any one time.

## Results

### Returns from proximity loggers

In total, 13 cattle and 12 badgers were fitted with data loggers and data were recovered for all individuals (Tables 3 & 4). One-second contacts made up a large proportion of recorded contacts (an average of 46% for badgers and 53% for cattle).

**Table 3:** Data recovered from badgers, and connectedness measures for intra-group (denoted *group*), inter-group (with individuals from neighbouring groups, denoted *neigh.*) and inter-species (denoted *cattle*) contacts: *C*_freq_, number of contacts/day and *C*_dur_, contact duration/day (in seconds), given as totals across the whole study.

ID	Group	Sex, Age	Days	All contacts	*C*_freq_ (*group*)	*C*_dur_ (*group*)	*C*_freq_ (*neigh.*)	*C*_dur_ (*neigh.*)	*C*_freq_ (*cattle*)	*C*_dur_ (*cattle*)
V53	Valley	F, A	79.8	4100	12.57	1484	0	0	0	0
V58	Valley	F, A	95.8	7284	24.43	2382	0	0	0.22	7.08
V59	Valley	M, Y	57.5	2642	17.31	1949	0	0	0.22	10.90
V61	Valley	F, Y	78.2	8485	32.54	4102	0.06	1.58	0	0
F62	Farm	M, Y	86.5	1206	14.24	1411	0.01	0.38	0	0
F63	Farm	F, A	75.1	1273	15.23	1623	0.02	0.78	0	0
C54	Cottages	F, A	22.9	1655	23.46	1494	n/a	n/a	n/a	n/a
C55	Cottages	F, Y	60.5	5894	35.84	2853	n/a	n/a	n/a	n/a
C56	Cottages	F, A	30.2	2184	30.65	2302	n/a	n/a	n/a	n/a
C57	Cottages	M, A	42.8	4501	50.74	5578	n/a	n/a	n/a	n/a
C60	Cottages	M, A	70.6	7433	48.61	4912	n/a	n/a	n/a	n/a
C64	Cottages	M, A	25.3	3640	57.21	5155	n/a	n/a	n/a	n/a
**Mean**			**60.4**	**4191**	**30.24**	**2937**	**0.02**	**0.46**	**0.07**	**3.00**

F, female; M, male; A, adult; Y, yearling.

**Table 4:** Data recovered from cattle, and connectedness measures for intra-herd and inter-species contacts: *C*_freq_, number of contacts/day and *C*_dur_, contact duration/day (in seconds), given as totals across the whole study.

ID	Days	All contacts	Intra-herd *C*_freq_	Intra-herd *C*_dur_	Inter-species *C*_freq_	Inter-species *C*_dur_
D1	41.4	1385	7.87	278	0	0
D2	52.2	7723	18.80	3687	0.01	0.18
D3	22.8	1207	26.37	1598	0	0
D4	39.9	2308	17.39	707	0.22	8.8
D5	52.1	2524	11.63	382	0	0
D6	18.4	1702	17.30	1348	0	0
D7	5.5	272	8.18	240	0	0
D8	40.3	1429	8.12	714	0	0
D9	22.9	1073	8.39	291	0	0
D10	22.9	876	7.05	250	0	0
D11	17.9	2456	19.57	12001	0.04	0.30
D12	22.9	1543	13.52	449	0	0
D13	17.9	1210	9.61	17155	0.06	0.57
**Mean**	**29.0**	**1978**	**13.37**	**3008**	**0.03**	**0.76**

Apart from large outlying values produced by cattle proximity loggers D11 and D13 for both *C*_dur_ and *AV*_dur_ (see Fig. S2A), the loggers recorded similar patterns of intra-specific interactions for both badgers and cattle, despite inherent differences which we expected to see between the two species in terms of their contact parameters. To distinguish between badgers and cattle, and to denote group membership, we prefix the ID numbers of dairy cattle with D and those of badgers with V (Valley), F (Farm) and C (Cottages).

Some loss of data due to non-recording of contacts became obvious when considering inter-group and inter-species contacts; although we obtained data on inter-group contacts between all six individuals from the Valley and Farm groups, only three of the proximity loggers actually recorded these interactions (V61, F62 & F63; Table 3). The logger of yearling male badger V59 only recorded contacts with two of the cattle (which were not reciprocated by the cattle loggers); instead, contacts with V59 were recorded on the loggers of another three cattle, implicating this badger in contacts with a total of five different cows.

### Badger intra-group connectedness and connectivity index (*CI*)

At the intra-group level, all individuals contacted each other, although to varying degrees. *C*_freq_ ranged from a minimum of 12.6 contacts/day, recorded at the Valley group, to a maximum of 57.2 contacts/day, recorded at the Cottages group (Table 3). *C*_dur_ ranged from just under 24 min/day at the Farm group to just over 1 h 30 min/day at the Cottages group (Table 3). The shortest *MAX*_int_ was two days, recorded at the Cottages group, while the longest *MAX*_int_ of just under seven days was recorded at the Valley group.

Both sampling period and social group had a significant effect on *C*_freq_ (LME: *F*_period_ = 5; d.f. = 3, 18, p<0.05; *F*_group_ = 9; d.f. = 2, 7, p<0.05; Table 5), but not on *AV*_dur_ or *MAX*_int_. *C*_freq_ was lower during sampling period 2 than during all other periods and more contacts were recorded in the Cottages group compared to Farm and Valley groups. Neither sex nor age had any effect on connectedness measures.

**Table 5:** Linear mixed-effects model for differences in the intra-group daily contact frequency (*C*_freq_), average duration per contact (*AV*_dur_) and maximum time interval between successive contacts (*MAX*_int_) for badgers.

Variable (df)	Model 1: *C*_freq_			Model 2: *AV*_dur_			Model 3: *MAX*_int_		
	Coeff.±s.e.	t	P	Coeff.±s.e.	t	P	Coeff.±s.e.	t	P
***Intercept*** (18)	1.27±0.11	11.96	<0.001***	2.01±0.05	44.5	<0.001***	0.62±0.09	6.65	<0.001***
***Period*** (18)									
2	−0.17±0.07	−2.56	0.020*	0.05±0.03	1.4	0.175	−0.04±0.10	−0.45	0.661
3	0.00±0.08	0.00	0.999	0.00±0.04	0.0	0.995	0.14±0.11	1.28	0.215
4	0.10±0.07	1.35	0.194	−0.03±0.04	−0.8	0.461	0.07±0.11	0.62	0.543
***Group*** (7)									
Cottages	0.32±0.11	2.77	0.028*	−0.11±0.05	−2.4	0.050	−0.17±0.08	−2.09	0.075
Farm	−0.22±0.15	−1.50	0.178	−0.04±0.06	−0.6	0.578	−0.06±0.12	−0.53	0.613
***Sex*** (7)									
Male	0.06±0.10	0.58	0.580	0.08±0.04	1.9	0.098	−0.14±0.08	−1.85	0.107
***Age*** (7)									
Yearling	0.03±0.11	0.25	0.808	0.01±0.04	0.1	0.918	−0.04±0.08	−0.52	0.622

Factors considered are: period (sampling period 1–4, see Table 2), group (Valley, Cottages, Farm), sex (female, male), age (adult, yearling). Results are shown relative to the reference category (period: 1; group: Valley; sex: female; age: adult); s.e., standard error; df, degrees of freedom.

*** P<0.001.

** P<0.01.

* P<0.05.

**Figure 2:**
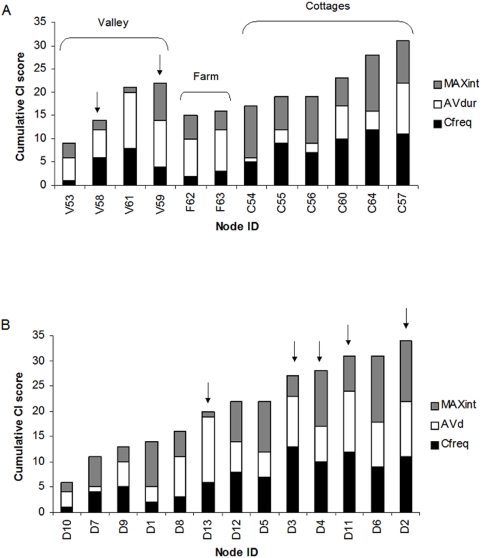
Connectivity Index (*CI*) scores. A) Badgers only (Cottages group included); B) Cattle only. Arrows mark individuals implicated in inter-species interactions.

Only *C*_freq_, *AV*_dur_ and *MAX*_int_ were included in the connectivity index *CI* (see supporting information), which is shown in Figure 2A. There was large variability in the different components of the *CI* score. Badgers from the Cottages group achieved higher scores for both *C*_freq_ and *MAX*_int_ compared to badgers from the Farm and Valley groups, although the latter achieved relatively higher scores for *AV*_dur_ (Fig. 2A).

### Cattle intra-herd connectedness and connectivity index (*CI*)

All but two individuals were in contact with each other, although to varying degrees (Table 4); no contacts were recorded between cows D2 and D9. *C*_freq_ ranged from a minimum of 7.1 contacts/day (D10) to a maximum of 26.4 contacts/day (D3). *C*_dur_ ranged from 4 min/day (D7) to nearly 5 h/day (D13). A minimum of 36 h (D6) and maximum of nearly eleven days (D13) was recorded for *MAX*_int_. Sampling period was not significant for all connectedness measures examined (Table 6).

**Table 6:** Linear mixed-effects model for differences in the intra-herd daily contact frequency (*C*_freq_), average duration per contact (*AV*_dur_) and maximum time interval between successive contacts (*MAX*_int_) for cattle.

Variable (df)	Model 1: *C*_freq_			Model 2: *AV*_dur_			Model 3: *MAX*_int_		
	Coeff.±s.e.	t	P	Coeff.±s.e.	t	P	Coeff.±s.e.	t	P
***Intercept*** (13)	0.89±0.11	8.33	<0.001***	1.50±0.17	9.03	<0.001***	0.27±0.07	3.61	0.003**
***Period*** (13)									
2	0.20±0.15	1.29	0.219	0.30±0.24	1.29	0.219	0.07±0.09	0.71	0.488
3	0.33±0.15	2.17	0.049*	0.04±0.24	0.18	0.862	0.10±0.09	1.13	0.280
4	0.14±0.15	0.94	0.363	0.50±0.23	2.21	0.045*	0.24±0.10	2.45	0.029*

The factor considered is period (sampling period 1–4, see Table 2). Results are shown relative to the reference category (period: 1); s.e., standard error; df, degrees of freedom.

*** P<0.001.

** P<0.01.

* P<0.05.

As for the badger connectivity index, only *C*_freq_, *AV*_dur_ and *MAX*_int_ were included in the connectivity index (see supporting information), and the index scores are shown in Figure 2B. Again there was large variability in terms of the different components of the *CI* score. The cattle with the highest *CI* scores were also the ones achieving the highest scores for *C*_freq_, while *AV*_dur_ and *MAX*_int_ were more variable throughout.

### Inter-group connectedness

**Figure 3:**
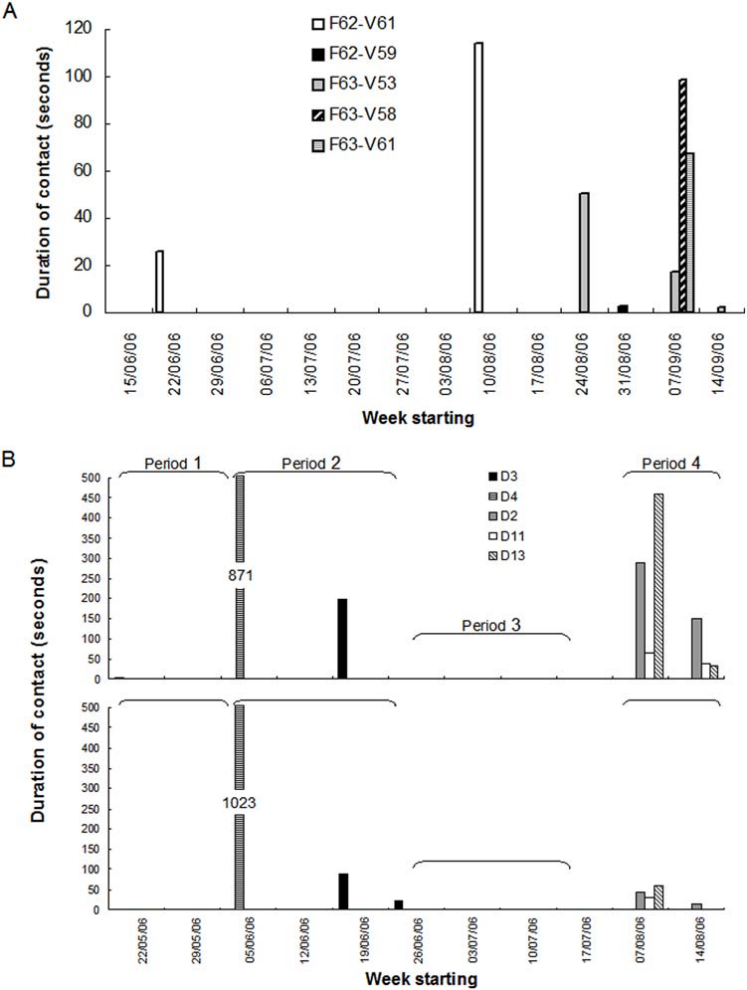
Contact profiles over time, showing the duration of contacts in seconds. A) inter-group contacts, B) inter-specific contacts (top: badger V58, adult female; bottom: badger V59, yearling male; weeks with no cattle collared are excluded). The data were summarised per week. Totals for the duration of contacts in the week starting 05/06/06 are shown. Note: the x-axis scale is not continuous, as weeks outside the sampling periods were omitted.

All six individual badgers from the Farm and Valley groups were involved in inter-group contacts. The inter-group contact profiles obtained from the three proximity loggers (V61, F62 & F63; Table 3) are shown in Figure 3A. A total of sixteen inter-group contacts were recorded by the data loggers throughout the study (four for V61, five for F62 and seven for F63), with an average duration of 32 seconds. Maximum *C*_freq_ and *C*_dur_ were calculated as 0.06 contacts/day and 1.6 seconds of contact/day, both recorded for yearling female V61 (Table 3). Maximum *AV*_dur_ was 1.8 seconds/day, recorded for F63. Female F63 contacted all three females of the Valley sett, while Farm male F62 contacted both a yearling male and a yearling female from the Valley group. The four contacts between F62 and Valley female V61 were recorded simultaneously on both proximity loggers, with an average duration per contact of 32 and 35 seconds respectively. Inter-group contacts happened infrequently and episodically: although F63 was collared from 29th June (by which time all Valley study badgers had already been collared) until the 19th September, all of her inter-group interactions recorded occurred between 30th August and 18th September, with all three Valley females being contacted on the night of the 11th September alone. Valley female V61 and Farm male F62 contacted each other once briefly in June, and then three times between the 10th and 14th of August.

### Inter-species connectedness

Six proximity data loggers (two badger loggers and four cattle loggers) recorded 103 and 32 inter-species interactions respectively (Tables 3 & 4). Overall, two Valley badgers and five cattle were implicated in inter-specific contacts, with the two badgers contacting all of the five cattle. All five cattle were in the top eight for *CI* rankings in cattle, with four out of the five amongst the top five (Fig. 2B). The resulting inter-species contact profiles for badgers V58 and V59, as recorded by the six data loggers, are shown in Figure 3B. No inter-specific contacts were recorded in sampling period 3, and only one short three-second contact was recorded in sampling period 1 (between V58 and D3). There was also much overlap in the timing of inter-specific contacts by the badgers, with contacts largely occurring within the same weeks.

## Discussion

Quantifying contact patterns in wildlife has been notoriously difficult, particularly where direct observation methods are impractical due to the elusive or nocturnal nature of a species. In this study, we employed novel proximity logging devices to quantify intra- and inter-specific interactions in a badger-cattle system. Our results represent the first continuous time records of wildlife-livestock contacts for a free-living wildlife-livestock disease system. In this study, we aimed to detect for the first time variability in contact patterns within and between species. Although the proximity loggers cannot achieve absolute spatial precision, they provide much better spatial resolution than radio-tracking studies [5], [23] and far more complete and less labour-intensive data collection than direct observation methods (e.g. see [31]), while the data only require a minimum of processing (e.g. deletion of one-second contacts) to achieve biologically meaningful records of inter-individual contacts [46].

Previous studies have documented good reciprocal recording between pairs of contact loggers, with respect to initiation time and contact duration, as well as the total number of contacts and total contact duration [24], [25]. In this study, we correlated the total number of contacts and total contact duration recorded between pairs of loggers in order to assess reciprocal recordings (i.e. whether the total number and duration of contacts corresponded well between pairs of loggers). Although there were some instances where contacts were recorded on only one of two ‘interacting’ loggers, overall correlations were significant, and discrepancies between pairs of loggers were generally caused by one collar recording a contact as one long event and the other as a series of shorter contacts, as has previously been observed [46].

Most individuals within groups were connected with each other, but both intra-group and intra-herd connectedness varied greatly among individuals and associations between individuals varied over time. A similar structure was recently shown in a species of tropical bat, where individuals often disassociated for short periods [48]. In beef systems, data loggers showed that the average number of daily contacts between unrelated cow and calf dyads ranged from 1–59 [25]. Similarly, we found variation in connectedness measures in both cattle and badgers. For example, the number of daily intra-group contacts ranged between 12–57 for badgers, while the number of daily intra-herd contacts ranged between 7–26 in cattle. For badgers, intra-group *AV*_dur_, the total amount of time, per day, spent in contact with other badgers, ranged from around 24 min (Farm) to just over 90 min (Cottages). This is surprising, given the fact that badgers often share nest chambers for day resting [49]. We regularly located our study animals at their day locations using radio-tracking, and found badgers sharing day resting sites on an average of only 30.1% of occasions; Cottages and Valley badgers shared resting sites most often (36.2% and 33.2% respectively), while the two Farm badgers were found in the same resting place on only 5.8% of occasions. This suggests that our study individuals spent much time resting away from others rather than sharing resting space. Furthermore, while all badgers from the same social group were directly in contact with each other, no interactions were recorded between two of the cattle, D2 (top of the *CI* rankings) and D9 (third from the bottom in the *CI* rankings). If high ranking and low ranking cows are overall less well-connected, low-ranking individuals may be at an inherently lower risk of catching an infection from another, higher ranking, individual within a herd.

In multi-host disease systems, where a pathogen can infect more than one of the species present, host species may combine to form a joint host community in which a pathogen can persist, depending on the extent of inter-specific interaction [50]. In terms of direct contacts, the two hosts in our wildlife-livestock system were mainly decoupled from each other, although episodic inter-species contact rates recorded by badger loggers exceeded interaction rates between neighbouring social groups in badgers (Table 3). In populations with strong spacing patterns, such as those caused by territoriality, disease establishment and persistence may be highly dependent on comparatively more frequent inter-species transmission instead of intra-species transmission [2]. This appears to be the case for the Valley badger group in our badger-cattle system. Here, the daily contact frequency and duration were higher in badger-cattle interactions than Valley badger group interactions; the true difference may be even greater due to the lower power settings and hence lower sensitivity employed by the cattle loggers.

Although nearly all of our cattle interacted with each other at some point in time and thus there were no true intra-specific cattle ‘hubs’ in the network (i.e. individuals with an extraordinarily large number of social contacts), there were differences in contact rates between individuals and four cattle out of the five most connected individuals were implicated in inter-species interactions. This suggests that some cattle, with higher intra-herd contact rates, are also more likely to be engaged in inter-specific interaction, and may constitute high-risk individuals for disease transmission, both within the cattle herd and between badgers and cattle. This corresponds with the work of Sauter & Morris [41], who showed that bTB reactor cattle were found within the top half of the cattle rank order. These reactors showed also the strongest interest in sedated possums, which were used to simulate the behaviour of terminally-ill tuberculous possums, the principal wildlife reservoir for bTB in New Zealand [41]. Whether or not a larger number of intra-herd contacts implies that cattle in our study were more sociable or simply had higher movement rates, leading them more regularly into contact with other cattle, remains unclear.

For bTB in Britain, indirect contacts between cattle and badgers via excretory products on pasture have been favoured traditionally over direct contacts as the main mode of bTB transmission to cattle. Our results show that, although direct badger-cattle interactions on pasture are relatively infrequent, they are unlikely to be as rare as previously thought [31]: five out of our 13 cattle came into close proximity with badgers over a six-month period. Farm buildings and feed stores have received considerable attention recently as places of contact (direct and indirect) between badgers and cattle [35], [36]. Our study has shown that direct badger-cattle contacts also occur on pasture. The focus of Defra's recommended farm biosecurity measures on preventing badger access to buildings and feed stores and avoiding indirect contacts with excretions in pasture [51] is therefore neglecting a potentially significant area of inter-species disease transmission. Quantifying the relative importance for transmission of direct and indirect contacts in different situations (field and on-farm) is an important area for further research, but it is clear that future bTB management strategies need to take account of all potential pathways for disease transmission.

Our data have demonstrated the heterogeneous nature of badger-cattle inter-specific contact networks. It is the types and rates of intra- and inter-specific contacts quantified in this study which drive bTB dynamics in host communities. As well as enhancing our understanding of likely patterns of disease spread in inter-specific host communities, these results also have significance for disease control; such quantification of contact rates can be used to inform and parameterise policy-led epidemiological models used to develop bTB control strategies in the UK. The predominance of specific individual cattle in inter-specific interactions with badgers, and hence with a higher risk of disease transmission to and from badgers, suggests that these individuals will act as ‘hubs’ in the inter-specific contact network. When considered alongside the heterogeneous pattern of cattle contact between farms, our results emphasise the potential benefits of more targeted cattle-bTB control regimes at both between- and within-farm levels. The current testing regimes recommended by Defra have failed to control bTB in cattle [26]. A higher frequency of bTB testing of highly connected markets and farms [17], combined with more frequent, targeted testing of dominant individuals within herds and a similarly targeted and therefore cost-effective application of any prospective cattle bTB vaccination programmes [52], [53], are likely to contribute to more effective and efficient strategies for controlling the disease.

## Supporting Information

Text S1 (0.03 MB DOC)

Figure S1 Significant correlations between intra-group connectedness measures for badgers. Daily contact frequency Cfreq is positively correlated with daily contact duration Cdur (A); average time interval between successive contacts AVint is negatively correlated with daily contact duration Cdur (B) and daily contact frequency Cfreq (C).
(0.06 MB TIF)

Figure S2 Significant correlations between intra-herd connectedness measures for cattle: daily contact duration Cdur is positively correlated with average contact duration AVdur (A); average time interval between successive contacts AVint is negatively correlated with daily contact frequency Cfreq (B).
(0.06 MB TIF)

## References

[1] Cleaveland S, Laurenson MK, Taylor LH (2001). Diseases of humans and their domestic mammals: pathogen characteristics, host range and the risk of emergence.. Philos Trans R Soc Lond B.

[2] Holt RD, Dobson AP, Begon M, Bowers RG, Schauber EM (2003). Parasite establishment in host communities.. Ecol Lett.

[3] Fenton A, Pedersen AB (2005). Community epidemiology framework for classifying disease threats.. Emerg Infect Dis.

[4] Keeling M (2005). The implications of network structure for epidemic dynamics.. Theor Pop Biol.

[5] Böhm M, Palphramand KL, Newton-Cross G, Hutchings MR, White PCL (2008). Dynamic interactions among badgers: implications for sociality and disease transmission.. J Anim Ecol.

[6] White PCL, Böhm M, Marion G, Hutchings MR (2008). Control of bovine tuberculosis in British livestock: there is no ‘silver bullet’.. Trends Microbiol.

[7] Ji W, White PCL, Clout MN (2005). Contact rates between possums revealed by proximity data loggers.. J Appl Ecol.

[8] Davidson RS, Marion G, Hutchings MR (2008). Effects of social hierarchy on disease persistence.. J Theor Biol.

[9] Woolhouse MEJ, Shaw DJ, Matthews L, Liu WC, Mellor DJ (2005). Epidemiological implications of the contact network structure for cattle and the 20–80 rule.. Biol Lett.

[10] Kao RR, Green DM, Johnson J, Kiss IZ (2007). Disease dynamics over very different time-scales: foot-and-mouth disease and scrapie on the network of livestock movements in the UK.. J R Soc Interface.

[11] Kiss IZ, Green DM, Kao RR (2008). The effect of network mixing patterns on epidemic dynamics and the efficacy of disease contact tracing.. J R Soc Interface.

[12] Turner J, Bowers RG, Clancy D, Behnke MC, Christley RM (2008). A network model of E. coli O157 transmission within a typical dairy herd: the effect of heterogeneity and clustering on the prevalence of infection.. J Theor Biol.

[13] Ball F, Mollison D, Scalia-Tomba G (1997). Epidemics with two levels of mixing.. Ann Appl Prob.

[14] Brennan ML, Kemp R, Christley RM (2008). Direct and indirect contacts between cattle farms in north-west England.. Prev Vet Med.

[15] Albert R, Jeong H, Barabási AL (2000). Error and attack tolerance of complex networks.. Nature.

[16] Woolhouse MEJ, Dye C, Etard J-F, Smith T, Charlwood JD (1997). Heterogeneities in the transmission of infectious agents: implications for the design of control programs.. Proc Nat Acad Sci USA.

[17] Kiss IZ, Green DM, Kao RR (2006). The network of sheep movements within Great Britain: network properties and their implications for infectious disease spread.. J R Soc Interface.

[18] Cowan DP (1987). Group-living in the European rabbit (*Oryctolagus cuniculus*): mutual benefit or resource localization?. J Anim Ecol.

[19] Day TD, O'Connor CE, Waas JR, Matthews LR (2000). Social interactions among captive brushtail possums (*Trichosurus vulpecula*).. Appl Anim Behav Sci.

[20] Totton SC, Tinline RR, Rosatte RC, Bigler LL (2002). Contact rates of racoons (*Procyon lotor*) at a communal feeding sites in rural Eastern Ontario.. J Wildl Dis.

[21] Buesching CD, Stopka P, Macdonald DW (2003). The social function of allo-marking in the European badger (*Meles meles*).. Behaviour.

[22] Doncaster CP (1990). Non-parametric estimates of interaction from radio-tracking data.. J Theor Biol.

[23] White PCL, Harris S (1994). Encounters between red foxes (*Vulpes vulpes*): implications for territory maintenance, social cohesion and dispersal.. J Anim Ecol.

[24] Prange S, Jordan T, Hunter C, Gehrt SD (2006). New radiocollars for the detection of proximity among individuals.. Wildlife Soc Bull.

[25] Swain DL, Bishop-Hurley GJ (2007). Using contact logging devices to explore animal affiliations: quantifying cow-calf interactions.. Appl Anim Behav Sci.

[26] ISG (2007). Bovine TB: The Scientific Evidence.

[27] Gilbert M, Mitchell A, Bourn D, Mawdsley J, Clifton-Hadley R (2005). Cattle movements and bovine tuberculosis in Great Britain.. Nature.

[28] Gopal R, Goodchild A, Hewinson G, de la Rua Domenech R, Clifton-Hadley R (2006). Introduction of bovine tuberculosis to north-east England by bought-in cattle.. Veterinary Record.

[29] Green DM, Kiss IZ, Mitchell AP, Kao RR (2008). Estimates for local and movement-based transmission of bovine tuberculosis in British cattle.. Proc R Soc Lond B.

[30] Gannon BW, Hayes CM, Roe JM (2007). Survival rate of airborne Mycobacterium bovis.. Res Vet Sci.

[31] Benham PFJ, Broom DM (1989). Interactions between cattle and badgers at pasture with reference to bovine tuberculosis transmission.. Brit Vet J.

[32] Muirhead RH, Gallagher J, Burn KJ (1974). Tuberculosis in wild badgers in Gloucestershire: epidemiology.. Vet Rec.

[33] Wilesmith J, Little TWA (1982). Bovine tuberculosis in domestic and wild animals in an area of Dorset I. Tuberculosis in cattle.. J Hyg.

[34] White PCL, Brown JA, Harris S (1993). Badgers (*Meles meles*), cattle and bovine tuberculosis (*Mycobacterium bovis*): a hypothesis to explain the influence of habitat on the risk of disease transmission in southwest England.. Proc R Soc Lond B.

[35] Garnett BT, Delahay RJ, Roper TJ (2002). Use of cattle farm resources by badgers (*Meles meles*) and risk of bovine tuberculosis (*Mycobacterium bovis*) transmission to cattle.. Proc R Soc Lond B.

[36] Garnett BT, Roper TJ, Delahay RJ (2003). Use of cattle troughs by badgers (*Meles meles*): a potential route for the transmission of bovine tuberculosis (*Mycobacterium bovis*) to cattle.. Appl Anim Behav Sci.

[37] Nolan A, Wilesmith JW (1994). Tuberculosis in badgers (*Meles meles*).. Vet Microbiol.

[38] Gallagher J, Clifton-Hadley RS (2000). Tuberculosis in badgers: a review of the disease and its significance for other animals.. Res Vet Sci.

[39] Gavier-Widen D, Chambers MA, Palmer N, Newell DG, Hewinson RG (2001). Pathology of natural Mycobacterium bovis infection in European badgers (*Meles meles*) and its relationship with bacterial excretion.. Vet Rec.

[40] Delahay RJ, Langton S, Smith GC, Clifton-Hadley RS, Cheeseman CL (2000). The spatio-temporal distribution of *Mycobacterium bovis* (bovine tuberculosis) infection in a high-density badger population.. J Anim Ecol.

[41] Sauter CM, Morris RS (1995). Dominance hierarchies in cattle and red deer (*Cervus elaphus*): their possible relationship to the transmission of bovine tuberculosis.. NZ Vet J.

[42] Palphramand KL (2005). Patterns of territoriality and space use in a moderate-density badger (*Meles meles*) population.

[43] Tuyttens FAM, Long B, Fawcett T, Skinner A, Brown JA (2001). Estimating group size and population density of Eurasian badgers *Meles meles* by quantifying latrine use.. J Appl Ecol.

[44] Harris S, Cresswell WJ, Cheeseman CL (1992). Age determination of badgers (*Meles meles*) from tooth wear: the need for a pragmatic approach.. J Zool.

[45] Sirtrack Ltd (2006). Sirtrack proximity logger information.

[46] Goodman EL (2007). Quantifying interactions in a high-density badger (*Meles meles*) population.

[47] Zuur AF, Ieno EN, Smith GM (2007). Analysing ecological data.

[48] Vonhof MJ, Whitehead H, Brock Fenton M (2004). Analysis of Spix's disc-winged bat association patterns and roosting home ranges reveal a novel social structure among bats.. Anim Behav.

[49] Roper TJ, Ostler JR, Schmid TK, Christian SF (2001). Sett use in European badgers *Meles meles*.. Behaviour.

[50] Bowers RG, Turner J (1997). Community structure and the interplay between interspecific infection and competition.. J Theor Biol.

[51] Defra (2007). Bovine TB Husbandry Working Group 2007. Advice on husbandry best practice.. http://www.defra.gov.uk/animalh/tb/abouttb/protect.htm.

[52] Defra (2005). Government strategic framework for the sustainable control of bovine tuberculosis (bTB) in Great Britain.

[53] Defra (2007). Options for vaccinating cattle against bovine tuberculosis.

