# Supplementary material for: Contact Networks in a Wildlife-Livestock Host Community: Identifying High-Risk Individuals in the Transmission of Bovine TB among Badgers and Cattle
Source: PLoS One. 2009 Apr 29;4(4):e5016. doi: 10.1371/journal.pone.0005016 (PMC2660423; doi:10.1371/journal.pone.0005016)
Supplement: Text S1 — (0.03 MB DOC) [file pone.0005016.s001.doc]

**Supporting Information**

Correlations of badger intra-group connectedness measures

*C*_dur_ was significantly correlated with *C*_freq_ and with *AV*_int_ (*C*_freq_: *r* = 0.926, d.f. = 10, p<0.001; *AV*_int_: *r* = -0.859, d.f. = 10, p<0.001; Fig S1A, S1B). *AV*_int_ was also significantly correlated with *C*_freq_ (*r* = -0.825, d.f. = 10, p<0.001; Fig. S1C). With an increasing daily contact duration, the number of contacts/day increased correspondingly (Fig. S1A), while the average time between successive contacts decreased (Fig. S1B). Consequently, the average time between successive contacts decreased with an increase in the number of contacts/day (Fig. S1C). As a result of this analysis, only *C*_freq_, *AV*_dur_ and *MAX*_int_ were included in the connectivity index for badgers.

### Correlations of cattle intra-herd connectedness measures

*C*_dur_ was highly significantly correlated with *AV*_dur_ (*r* = 0.960, d.f. = 11, p<0.001; Fig. S2A). *C*_freq_ was significantly correlated with *AV*_int_ (*r* = -0.707, d.f. = 11, p<0.01; Fig. S2B). Average contact duration increased with daily contact duration (Fig. S2A), while the average time between contacts decreased with an increase in the number of contacts/day (Fig. S2B). As for the badgers above, only *C*_freq_, *AV*_dur_ and *MAX*_int_ were included in the cattle connectivity index as a result of this analysis.
