# Supplementary figures and images for: Contact Networks in a Wildlife-Livestock Host Community: Identifying High-Risk Individuals in the Transmission of Bovine TB among Badgers and Cattle
Source: PLoS One. 2009 Apr 29;4(4):e5016. doi: 10.1371/journal.pone.0005016 (PMC2660423; doi:10.1371/journal.pone.0005016)

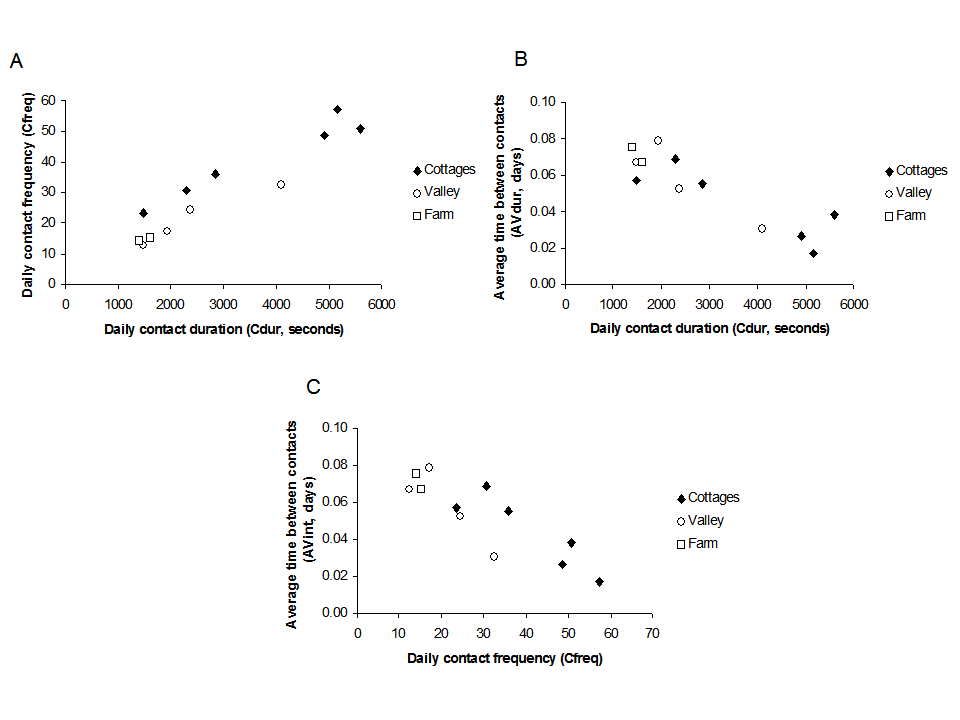

Supplement: Figure S1 — Significant correlations between intra-group connectedness measures for badgers. Daily contact frequency Cfreq is positively correlated with daily contact duration Cdur (A); average time interval between successive contacts AVint is negatively correlated with daily contact duration Cdur (B) and daily contact frequency Cfreq (C). (0.06 MB TIF) [file pone.0005016.s002.tif]

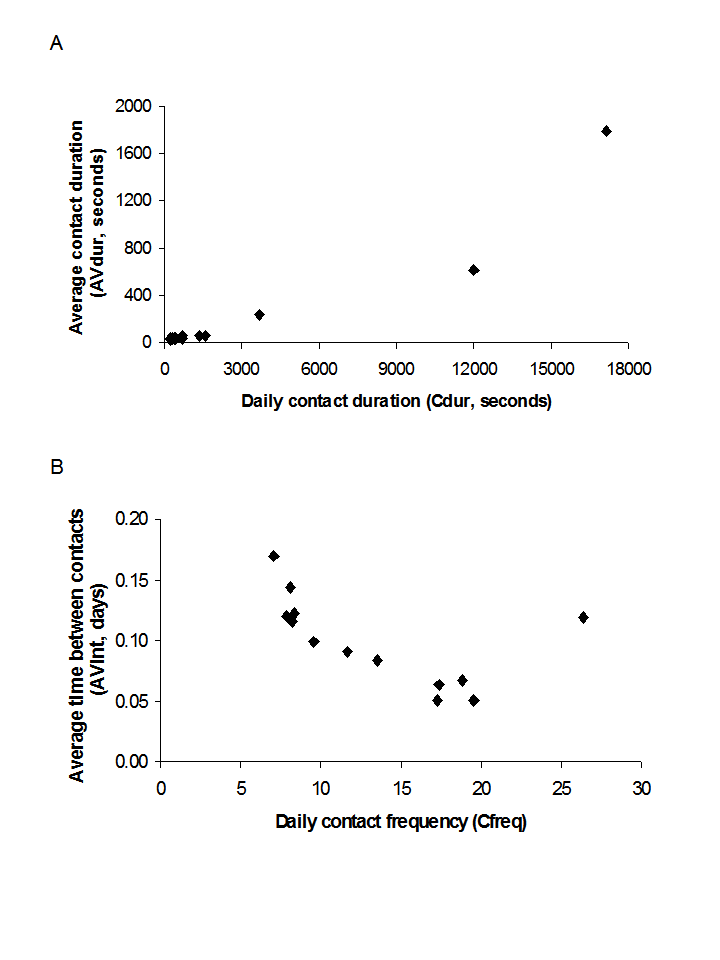

Supplement: Figure S2 — Significant correlations between intra-herd connectedness measures for cattle: daily contact duration Cdur is positively correlated with average contact duration AVdur (A); average time interval between successive contacts AVint is negatively correlated with daily contact frequency Cfreq (B). (0.06 MB TIF) [file pone.0005016.s003.tif]
